# Diet Supplementation with a Bioactive Pomace Extract from *Olea europaea* Partially Mitigates Negative Effects on Gut Health Arising from a Short-Term Fasting Period in Broiler Chickens

**DOI:** 10.3390/ani10020349

**Published:** 2020-02-22

**Authors:** Javier Herrero-Encinas, Marta Blanch, José J. Pastor, David Menoyo

**Affiliations:** 1 Departamento de Producción Agraria, Universidad Politécnica de Madrid, ETS Ingeniería Agronómica, Alimentaria y de los Biosistemas, 28040 Madrid, Spain; j.herreroe@alumnos.upm.es; 2 Lucta S. A., Innovation Division, UAB Research Park, Edifici Eureka, 08193 Bellaterra, Spain; marta.blanch@lucta.com (M.B.); jose.pastor@lucta.com (J.J.P.)

**Keywords:** broiler chicken, olive pomace extract, short-term fasting, intestinal permeability, gut health, anti-inflammatory

## Abstract

**Simple Summary:**

Plant-derived feed additives have been gaining interest as a means to maintain gut health in poultry. Recent studies have shown that fasting broilers up to 24 h triggers intestinal permeability increase and might be used as an experimental model to challenge gut health. The present study has demonstrated that feeding broiler chickens with an olive pomace extract rich in bioactive anti-inflammatory compounds do not negatively affect growth performance. Moreover, the olive pomace extract reduced some of the negative effects that a short-term fasting period induced in the intestine of broiler chickens.

**Abstract:**

The effects of supplementing chicken diets with an olive pomace extract (OE) from *Olea europaea* on performance and gut health after a challenge of intestinal permeability (IP) increase were studied. Treatments included a control diet with no additives (CF), and diets supplemented with 100 ppm of monensin (MF) or with 500 (OE500F) and 1500 ppm (OE1500F) of an OE. At 14 d, all birds, except those allocated in a control group (CNF), were submitted to a 15.5 h short-term fasting period to induce IP increase. Fasting increased (*p* < 0.05) lactulose/mannitol ratio and Alpha 1 Acid Glycoprotein concentration, and reduced (*p* < 0.001) villus/crypt ratio. Moreover, a down-regulation of Claudin-1 (*p* < 0.05), an up-regulation of TLR4 and IL-8 (*p* < 0.05) ileal gene expression was observed in CF birds compared to CNF. OE500F treatment reduced duodenal crypt depth compared to CF (*p* < 0.05; OE linear effect). Mannitol concentration and ileal IL-8 expression were reduced in OE500F compared to CF and OE1500F (*p* = 0.05). Fasting challenge induced an increase in IP triggering an inflammatory response. Supplementation of OE up to 1500 ppm did not affect growth performance and alleviated some of the negative effects of the fasting challenge.

## 1. Introduction

The current need to find alternatives to the use of antibiotics in poultry feeds is opening opportunities for feed additives such as phytochemicals [1,2]. Phytochemicals are a heterogeneous group of substances, including aromatic plants, volatile compounds (essential oils) and plant extracts, which exert the desired effects on the intestinal health because of their content of secondary metabolites (e.g., polyphenols, terpenes) [3]. Diverse combinations of natural alternatives have shown to promote growth and enhance gut health, but their mechanisms of action are not fully understood [2]. Moreover, bioactive phytochemicals can stimulate innate immunity and might be an alternative to control coccidiosis in poultry [4]. According to Gadde et al. [5], antibiotic alternatives should be developed on the antibiotic mechanism of action to achieve non-antibiotic surrogates. In this regard, Niewold [6] suggested that antibiotic substances fed at a sub-therapeutic dosage reduce the host inflammatory response by decreasing pro-inflammatory cytokine production. Thus, antibiotics generate energy saving from immune function and, consequently, promote animal growth [6,7].

By-products from olive oil mill processing are rich in bioactive substances, including polyphenols, triterpenic acids and oleuropeosides, with anti-inflammatory and antioxidant properties [8,9,10]. During the last years, many studies have focused on the utilization of these by-products in poultry feeding, showing positive effects in broiler chickens’ growth performance and antioxidant capacity [11,12], or enhancing egg weight, yolk index, intestinal absorption capacity with no negative effects on laying hens’ performance [13,14]. Moreover, several authors have tested different olive pomace extracts in broiler chickens, pigs, cattle and sea bream, showing positive results in intestinal health, gut integrity and innate immune functions [15,16,17,18]. 

Intestinal barrier plays an important role in nutrient digestion, and gut barrier disturbances, such as microbial dysbiosis, leaky gut, and inflammation of the mucosal tissue, alter health and performance in poultry [19,20]. In this context, a short-term fasting period (<24 h) has been shown to increase the intestinal permeability in broiler chickens and might be a good model to test dietary effects on gut barrier functions [21,22]. The present study aims to investigate the effects of supplementing broiler chickens’ diets with a bioactive olive pomace extract (OE) from *Olea europaea* on growth performance, and whether it will help to maintain gut health after a challenge of intestinal permeability increase induced by a short-term fasting period. The rationale behind this study is that bioactive compounds of the OE have barrier function-enhancing activities when intestinal permeability increases.

## 2. Materials and Methods 

### 2.1. Housing and Experimental Animals

The feeding trial was carried out at Universidad Politécnica de Madrid experimental facilities (Agricultural Production Department, Madrid). All experimental procedures were accepted by the animal Ethics Committee of the Universidad Politécnica and Comunidad de Madrid (reference: PROEX 007/15) in compliance with the Spanish Guidelines for the Care and Use of Animals in Research [23]. 

The trial was conducted on 660 one-day-old male broiler chicks (Cobb 500) from a commercial hatchery (Incubadora Uvesa, Tudela, Navarra, Spain). Animals were randomly assigned to 30 floor pens (1.1 × 1.1 m) with 22 chickens per pen (6 pen/treatment × 5 treatments) provided with a hopper feeder, bell drinker and bedded with wood shaving. The facility temperature was kept at 34 °C during the first week of age and gradually decreased (at rate of 2 °C per week) to 26 °C at 5 weeks of age. Humidity and ventilation were automatically controlled. The lighting program started with 24L:0D during the first week and 18L:6D light afterwards. 

### 2.2. Diet and Experimental Design

The feeding program consisted of a starter feed (23.0% of crude protein (CP) and 2974 kcal/kg of apparent metabolizable energy (AME)), fed in crumbles from 1 to 21 d of age; and a grower feed (CP = 21.8%, AME = 3088 kcal/kg), fed from 21 to 32 d of age as pellets with 3 mm diameter. The composition of the starter and grower basal diets is shown in Table 1. The basal diet with no medication and no additives was used as a negative control. The other three diets were obtained by adding on top of the basal diet 500 (OE500F) or 1500 ppm (OE1500F) of an olive pomace extract provided by Lucta S. A. (Madrid, Spain), or 100 ppm of monensin (MF) (Elanco Valquimia S. A.) and used as a positive control. Supplemented OE was a standardized olive pomace extract containing ≥10% of triterpenes and ≥2% of polyphenols, quantified by HPLC-UV as oleanolic acid and hydroxytyrosol equivalents, respectively. Diets were manufactured at IRTA (Mas de Bover, Constantí, Spain).

At the beginning of the trial, chickens were divided at random into 5 groups. One group was fed the control diet ad libitum throughout the feeding trial, from 1 to 32 d of age (treatment control no fasted, CNF). The other 4 groups were also fed ad libitum from 1 to 32 d of age except for a short-term fasting period (F) of 15.5 h at 14 d of age. They received the control diet (treatment control and fasted, CF) or any of the supplemented diets (OE500F, OE1500F and MF).

### 2.3. Short-Term Fasting Period and Intestinal Permeability Study

From 1 to 14 d of age, animals were fed their respective experimental diets. At day 14, birds from all treatments, except those of CNF, were feed deprived for 15.5 h. Intestinal permeability was measured by the lactulose/mannitol (L/M) test following the protocol described by Gilani et al. [22]. To this end, following the 15.5 h fasting period (15 d of age), two birds per pen (12 chicks per treatment) were randomly selected and administered an oral gavage of lactulose and mannitol. Lactulose and mannitol solution contained 25 g lactulose and 5 g of mannitol (Sigma Aldrich, Alcobendas, Madrid, Spain) dissolved in 100 mL Milli-Q water at 25 °C. Birds were slaughtered (CO_2_ atmosphere asphyxiation) and sampled 90 min after the oral gavage. After this short-term fasting period, 16 chickens per pen were fed their respective diets ad libitum until the end of the trial at 32 d of age.

### 2.4. Experimental Diet Analysis

The experimental feed was analyzed following the standard methods of AOAC [24] for dry matter (934.01), ether extract (920.39), total ash in muffle (942.05) and crude protein by combustion method (968.06) using FP-528 nitrogen analyzer (LECO^®^, St. Joseph, MI, USA). The gross energy was analyzed by adiabatic bomb calorimeter (PARR, 1356 model, Parr Instrument Company, Moline, IL, USA).

### 2.5. Productive Traits and Sampling

To calculate the average daily gain (ADG), average daily feed intake (ADFI) and feed conversion ratio (FCR), body weight and feed intake were recorded per pen at 7, 14, 21, 28 and 32 d of age. At 15 d of age (after the 15.5 h fasting period), the two birds per pen randomly selected for the intestinal permeability study were slaughtered by asphyxiation in CO_2_ and sampled. For gene expression analysis, 200 mg of ileal mucosal scraping were taken in RNA later (Invitrogen, Carlsbad, CA) following the manufacturer’s instructions, and further stored at −80°C. For intestinal morphology, 3 cm length segments for the middle part of duodenum were collected and stored in 10% neutral buffered formaldehyde solution. To obtain the plasma, blood samples were collected immediately post-mortem via cardiac puncture into tubes containing EDTA and aprotinin (BD Vacutainer^®^, Plymouth, UK), held in ice for 30 min, centrifuged at 2000× *g* for 10 min and stored at −80 °C to later analyze alpha-1-acid glycoprotein (AGP), cytokines and lactulose and mannitol concentration. At the end of the trial (32 d of age), remaining animals were slaughtered by asphyxiation in CO_2_ atmosphere.

### 2.6. Plasma Lactulose and d-Mannitol Concentrations Analysis by Ultra-Performance Liquid Chromatography (UPLC)

Determination of plasma lactulose and d-mannitol concentrations were performed in an Acquity UPLC (Waters Corp., Mildford, MA, USA) connected to a Xevo-G2 Qtof mass spectrometer (Waters Corp., Mildford, MA, USA) operating in full scan negative mode (100 to 1200 m/z). Chromatographic separation was achieved with a linear gradient using a BEH amide column (21 × 100 mm, 1.7 µm, Waters Corp., Mildford, MA, USA) and mobile phases comprising A = 10 mM ammonium acetate in ACN:H_2_O (9:1) and B = 10 mM ammonium acetate in ACN:H_2_O (4:6). Flow rate was set to 0.5 ml/min and oven temperature to 40 °C. Leucine-enkephalin (200 ng/ml) was used as lock mass. Data processing was performed with QuanLynx software (Waters Corp., Mildford, MA, USA). Ion areas were used for quantification based on standard curves prepared using authentic standards and raffinose as internal standards (IS). Plasma samples (25 µL) were submitted to protein precipitation with 56 µL of IS solution. Four independent replicates per sample were prepared and analyzed.

### 2.7. Cytokine and Alpha 1 Acid Glycoprotein (AGP) Plasma Concentration

Cytokine quantification in plasma was performed with commercial ELISA according to manufacturer’s instructions (IL-1β, SEA565Ga; IL-8, SEA080Ga, Wuhan USCN Business Co. Ltd., Wuhan, China). Kit standards and test samples optical densities were read at 450 nm by an ELISA plate reader (Sunrise Microplate Reader, Tecan Trading AG, Switzerland). AGP plasma concentration was measured using ELISA Kit (ab157690; Abcam, Cambridge, MA, USA) according to the manufacturer’s instructions.

### 2.8. Gene Expression Analysis

Total RNA was extracted from approximately 50 mg of ileal mucosal scraping with TRIzol reagent (Invitrogen, Carlsbad, CA, USA), disrupted with a mixer mill MM-400 (Retsch, Stuttgart, Germany) and isolated by using the GenElute Mammalian Total RNA Miniprep Kit (Sigma-Aldrich Corporation, St. Louis, MO, USA). To prevent genomic DNA contamination, an “in column” DNase step was performed by using the RNase-Free DNase Set (Quiagen, Australia). Extracted RNA yield and quality were measured by spectrophotometry (EpochTM, BioTek, Winoosky, VT, USA) combined with the Take3TM Micro-Volume Plate (BioTek, Santa Barbara, CA, USA) by absorbance at wavelengths of 260 and 280 nm. Reverse transcription of around 2400 ng of extracted RNA was performed with the SuperScript VILO Master Mix (Invitrogen, Carlsbad, CA, USA). The quantitative Real-Time PCR (qRT-PCR) analysis was performed in a 7300 Real Time PCR System (Applied Biosystems, Foster City, CA, USA) with already tested and published primer conditions. To assay target genes, primers were obtained from the literature (Table 2). Target genes were selected according to their functional roles as: tight junction proteins (Claudin-1 and 3), enterocyte protection (HSP-70), immune function (TLR-2β, TLR4, TGF-β4, IL-8, and Bu-1) and carbohydrate (ChREBP) and lipid (FABP6) metabolism. To quantify the relative gene expression, UB and β Actin were used as housekeeping genes.

Samples were analyzed in triplicate using the right amount of each primer, ultra-purified water and SYBR^®^ Green Master Mix (Applied Biosystems, Foster City, CA, USA).

### 2.9. Intestinal Morphology Analysis

Samples collected at 15 d of age were embedded in paraffin using a tissue processor. Sections of 2.5 µm were stained with hematoxylin and eosin analyzed with an Olympus BX-40 (Olympus Optical Co., Ltd., Tokyo, Japan) digital camera and the Soft software version 3.2 C 4040 Z (Soft Imaging System, Olympus, GmbH, Hamburg, Germany). Images were analyzed eye blinded by the same person. Villus height (VH) and crypt depth (CD) of 9 intact villi per section were recorded for each animal.

### 2.10. Statistical Analysis

Data were analyzed using SAS [33]. The data were checked for normality distribution by using the Shapiro–Wilk’s test and homogeneity of variance was established by using the Levene’s test. Diets were the main fixed effect in the model. For performance analysis, the experimental unit was the pen, and for the intestinal morphology, lactulose and mannitol plasma concentration and gene expression analysis, the animal was the experimental unit. The effect of short-term fasting on the studied variables was determined by a contrast comparing CNF and CF groups. Polynomial contrasts were used to test the linear and no linear effect of OE inclusion. The positive control group (MF) was compared with the rest of the fasted groups by using the Dunnett’s test at α = 0.05. Results are presented in tables as mean and the standard error of means (SEM). For genes displaying efficiency different from 2 (E ≠ 2), Ct values were adjusted according to the previous model described by other authors [34].

## 3. Results

### 3.1. Daily Gain, Feed Intake, and Feed Conversion Ratio

No significant differences were observed in ADG, ADFI and FCR among treatments before fasting period (from 1 to 14 d of age), being on average 31.7 g/bird/d, 37.4 g/bird/d and 1.20 g/g, respectively. After the fasting period the ADG of CF birds tended (*p* = 0.06) to be lower compared to CNF from 14 to 21 d of age. In the last period, from 21 to 32 d of age, no significant differences in performance were observed among groups (Table 3).

### 3.2. Lactulose and Mannitol Concentration and L/M Ratio

Plasma mannitol concentration was significantly (*p* < 0.001) reduced and plasma lactulose and L/M were significantly (*p* < 0.001) increased in CF birds compared to CNF on day 15 (Table 4).

No significant differences were observed on lactulose or the L/M plasma concentration among fasted treatments. However, a non-linear trend (*p* = 0.053) on plasma mannitol was observed with the inclusion of OE in the diet, showing higher concentration in birds fed OE500F diet compared to CF and OE1500F groups.

### 3.3. Cytokine and AGP Plasma Concentration

No differences in IL-1β and IL-8 plasma concentration were observed among treatments (Table 5). However, AGP plasma concentration significantly (*p* < 0.05) increased as a consequence of fasting (CNF vs. CF treatments). Moreover, CF treatment showed significantly (*p* < 0.05) higher AGP plasma concentration compared to MF treatment.

### 3.4. Gene Expression

Gene expression data are shown in Table 6. The expression of Claudin3, TLR-2β, TGF-β4, HSP-70 and Bu-1 in the ileum was not affected by a short-term fasting period. However, tight junction Claudin1 gene expression was significantly down-regulated (*p* < 0.05) in fasted birds. Moreover, the expression of FABP6 was significantly (*p* < 0.01) and that of ChREBP tended (*p* < 0.10) to be down-regulated in fasted birds. By contrast, the expression of TLR4 and IL-8 were significantly up-regulated (*p* < 0.05) in fasted chickens.

Among fasted treatments, a non-linear tendency (*p* = 0.053) of ileal IL-8 expression was observed with OE inclusion in the diet, displaying birds fed the OE500F diet lower expression levels than those of the CF and the OE1500F groups. Furthermore, the expression of Bu-1 linearly increased (*p* < 0.05) with OE inclusion levels.

### 3.5. Histology

Results of intestinal morphology of duodenum are shown in Table 7. Control fasted birds showed significantly (*p* < 0.001) higher CD and lower villus height/crypt depth ratio (V/C) compared to CNF treatment. 

Among fasted treatments, the MF group showed significantly shorter VH compared to the CF and OE500F treatments (*p* < 0.05), but similar than those on the OE1500F treatment. The inclusion of OE decreased (*p* < 0.05), linearly, VH and CD.

## 4. Discussion

### 4.1. Effect of Fasting Period

In the present study birds fasted for 15.5 h at 14 d of age showed similar ADFI and FCR from 14 to 21 d of age than no fasted birds; however, the ADG tended to be lower in fasted birds. From 21 to 31 d of age, short-term fasted and no fasted showed similar performance. Chamblee et al. [35] observed a rapid compensatory growth in birds upon refeeding after short-term (12 h) fasting period. Modern fast growing broiler breeds might need more time to fully compensate growth performance after a short-term (less than 24 h) fasting period.

Intestinal health is determined by factors such as nutrition, microbiome and environment and their effects on the host immunity and mucosal barrier permeability [20]. The latter has been proven to be impaired when broiler chickens are short-term fasted (less than 24 h), as measured by FITC-d, lactulose, mannitol and L/M ratio markers [21,22]. Lactulose is a high-molecular-weight sugar that is absorbed via enterocyte tight junctions (paracellular transport) and mannitol is a lower-molecular-weight sugar absorbed straight into the blood (transcellular transport) [21,22,36]. In the present study, the concentration of mannitol significantly decreased and that of lactulose and L/M increased in the plasma of birds after a 15.5 h fasting period, confirming the detrimental effects on intestinal permeability observed in chickens after a short-term fasting period [22]. The observed increment in the paracellular transport of fasted animals was associated with a decreased expression of Claudin-1 but no Claudin-3 in the ileum. Claudins are important structural components of the tight junctions and Claudin-1 and -3 are directly involved in the paracellular permeability [37]. In a recent study, Gilani et al. [30] reported a decreased expression of Claudin-3 but no effect on Claudin-1 and other tight junction proteins in short-term fasted birds. Despite the fact that a decreased expression of Claudin-1 has been related with an increased permeability in intestinal disorders, there seems not to be a clear pattern for Claudin-3 expression, which may or may not be affected [38]. On the other hand, fasting period significantly reduced FABP6 expression in the ileum, corroborating results showed by Gilani et al. [30]. This protein is involved in bile acid transport across the epithelium, and, according to Gilani et al. [30], FABP6 downregulation in fasted birds might indicate a reduced bile acid production as a consequence of dietary fatty acid absence. Furthermore, considering the described effect of several dietary components (e.g., glutamine, fatty acids) and bacteria on claudin expression [37], it seems plausible that the lack of nutrients during fasting might affect Claudin-1 expression and increase permeability.

An increase in intestinal permeability leads to an increase of food and bacterial antigens that might trigger an inflammatory response [39]. Birds feed deprived for 15.5 h significantly upregulated TLR4 expression in the ileum but not that of TLR2-β. Both are regulators of the innate immune system that induce an inflammatory response after detecting pathogenic and non-pathogenic molecules [27]. LPS produced by gram-negative bacteria or dietary fatty acids have shown to activate TLR4 and the inflammatory response after a reduced integrity of the intestinal barrier in obesity models [40]. Moreover, IL-8 gene expression was also upregulated in the ileum after the fasting period. This chemokine recruits heterophils at a local level in response to the presence of bacteria or inflammatory cytokines [27,41]. A higher IL-8 expression related to the inflammatory response was observed in a gut barrier failure model with diet and coccidiosis [27]. In addition, the expression of the anti-inflammatory cytokine TGF-β4, and that of HSP-70 included in tissue protection and repair were not affected by the short-term fasting. This is in agreement with Gilani et al. [30], who showed the absence of changes in HSP-70 gene expression in the jejunum and ileum of up to 19.5 h fasted chickens. Taken together with these results, it seems that feed withdrawal for 15.5 h induced an intestinal inflammatory response through TLR4 signaling.

Broilers fasted during 15.5 h showed higher AGP serum concentration compared to CNF animals. Similar findings were observed by Najafi et al. [42] in broilers after a longer period of food deprivation (30 h). This acute phase protein has been also reported to increase in serum of birds, with an increase of intestinal permeability caused by a rich NSP diet plus a coccidian challenge [27]. Therefore, it is plausible that the high AGP values reported in this study are closely related to the observed intestinal inflammation and permeability increase and/or the stress induced by feed deprivation. 

Studies in broilers have addressed the effects of a short-term (<24 h) fasting period on intestinal morphology by measuring VH and CD [30,43]. Changes in intestinal morphology seem to be related to the hours of feed withdrawal and to the intestinal section, with duodenum and jejunum responding faster than ileum [30,43]. Higher VH and CD have been reported in the jejunum or in the ileum of short-term fasted birds [43]. According to Thompson and Applegate [43], increased CD in short-term fasted birds suggests a need of higher tissue turnover to maintain VH in order to maximize absorption when nutrients become available again. However, there is no clear relationship between such changes and intestinal permeability increase [30,43]. In this study, increase of intestinal permeability in fasted birds was associated with unaltered VH but significant augmented CD in duodenum. Similar results were observed by Chen et al. [27] in chickens at 28 d of age, with intestinal permeability increase caused by a rich NSP diet plus a coccidial challenge. Duodenal samples of challenged birds showed similar VH but higher CD in an attempt to enhance cell proliferation to maintain gut barrier integrity [27]. If short-term fasting effects on VH and CD are a consequence to maintain the epithelial structure, to maximize nutrient absorption or to maintain gut integrity deserves future studies.

### 4.2. Effect of Olive Pomace Extract

The results of this study showed that supplementation of different levels of OE or with M did not alter chicken performance during the different phases of the experiment. This is in agreement with Leskovec et al. [44] in broiler chickens fed diets supplemented with an olive leaf extract. Furthermore, King et al. [45] reported no significant differences in broiler performance after supplementing an olive pomace extract in water. By contrast, Herrero-Encinas et al. [17] were able to detect higher ADG and better FCR in broiler chickens fed 1000 ppm of a bioactive extract from *Olea europaea* from 35 to 42 d of age. Moreover, utilization of olive leaf extracts in broilers’ diet improved FCR and body weight in the study of Sarica and Ürmez [46]. Thus, the potential beneficial effects of the bioactive compounds of extracts from *Olea europaea* on productive parameters might depend on the composition and concentration of the bioactive substances, bird age, mode of supplementation (water vs. feed). 

Among fasted birds, the significantly higher mannitol concentration was noticeable in the plasma of birds fed the OE500F, compared to those fed the CF or the OE1500F. These results are in agreement with Liehr et al. [16], who showed an intestinal functionality improvement by an increment of plasma mannitol concentration in pigs fed an olive oil extract and chronically challenged with lipopolysaccharides. Therefore, the higher mannitol concentration observed in birds supplemented with 500 ppm of OE in our study could be related to a lower transcellular transport damage when they were short-term fasted. However, the concentration of lactulose in the plasma and the expression of Claudin-1 and -3 and FABP6 in the ileum were similar among dietary treatments after 15.5 h of feed withdrawal. This might be indicative of a lack of effect of OE on paracellular transport increase after the short-term fasting period. 

Bioactive compounds of olive pomace have shown immunomodulatory effects in broiler chickens, pigs, calves and fish by reducing the production of pro-inflammatory cytokines and increasing the anti-inflammatory cytokines [15,16,17,18]. Interestingly, broiler chickens fed OE500F showed a significant down-regulation of ileal IL-8 expression, compared with birds in the CF and OE1500F. Moreover, the inclusion of OE increased Bu-1 expression, suggesting a stimulatory effect on humoral immunity. An immunosuppressant effect on IL-8 and a stimulatory effect on Bu-1 have been observed in our previous study with broilers fed an olive pomace extract in the growing phase [17]. Furthermore, increased B-cell markers has been previously described in broiler chickens fed plant extracts rich in polyphenolic compounds [47]. This immunomodulatory effect of OE deserves future research in a context of experimentally-induced disease. 

Among dietary treatments, birds fasted and fed MF and OE1500F diets showed similar VH, but significantly lower than those fed the CF and OE500F. Moreover, the increase of OE in diets linearly decreased CD. This is in agreement with other studies of broiler chickens, that have shown reduced jejunal CD in birds fed with garlic extract compared to a control [48]. According to these authors, a reduced CD suggests a lower turnover rate to maintain VH and, hence, lower energy needs to maintain gut integrity. In the present study, this might be the case with birds fed the OE500F, as they seem to maintain VH with lower CD. However, it was not observed with the highest dose of the extract.

## 5. Conclusions

Under the conditions of the present study, the inclusion of up to 1500 ppm of an extract rich in bioactive compounds from *Olea europaea* in starter and grower broiler diets did not affect performance. The 15.5 h fasting period significantly increased intestinal permeability triggering an inflammatory response mediated by TLR4. The inclusion of 500 ppm of the OE attenuated some of the negative effects of increased intestinal permeability associated to this short-term fasting challenge.

## Figures and Tables

**Table 1:** Composition of starter experimental diets (%, as fed basis, unless otherwise indicated) of experimental diets.

Item ^1^	Starter Diet	Grower Diet
Ingredient composition		
Maize	54.28	56.41
Soybean meal (48% CP)	38.43	35.73
Soybean oil	3.50	1.44
Animal fat	-	3.00
Dicalcium phosphate	1.82	1.74
Calcium carbonate	0.76	0.71
Salt	0.40	0.38
DL-methionine	0.29	0.22
L-lysine HCl	0.13	0.03
L-threonine	0.02	-
Minerals and Vitamins ^2^	0.30	0.30
Choline chloride	0.05	0.02
Noxyfeed ^3^	0.02	0.02
Calculated composition		
AME, Kcal/Kg	2974	3088
Crude protein	23.0	21.8
Ash	6.30	5.90
Ether extract	6.10	7.10
Lysine	1.29	1.20
Methionine	0.62	0.55
Methionine + cysteine	0.97	0.90
Threonine	0.84	0.82
Tryptophan	0.25	0.25
Calcium	0.95	0.90
Total phosphorus	0.70	0.67
Non phytate phosphorus	0.45	0.43
Sodium	0.17	0.16
Analyzed composition (% DM)		
Dry matter	88.4	89.2
Ash	6.30	6.10
Crude protein	23.8	21.2
Ether extract	6.94	7.50
Gross energy (Kcal/kg)	4030	4082

^1^ CP, crude protein; AME, apparent metabolizable energy; DM, dry matter. ^2^ The premix provides per kg feed: Vitamin A (E672) 10000 UI; vitamin D_3_ (E671) 4800 UI; vitamin E (alfa tocopherol) 45 mg; vitamin B_1_ 3 mg; vitamin B_2_ 9 mg; vitamin B_6_ 4.5 mg; vitamin B_12_ 40 μg; vitamin K_3_ 3 mg; calcium pantothenate 16.5 mg; nicotinic acid 51 mg; folic acid 1.8 mg; biotin 0.15 mg; Fe (E1) (from FeSO_4_·H_2_O) 54 mg; I (E2) (from Ca(IO_3_)_2_) 1.2 mg; Cu (E4) (from CuSO_4_·5H_2_O) 12 mg; Mn (E5) (from MnO) 90 mg; Zn (E6) (from ZnO) 66 mg; Se (E8) (from Na_2_SeO_3_) 0.18 mg. ^3^ ITPSA, Barcelona, Spain. Contains BHT+ propyl gallate (56%) and citric acid (14%).

**Table 2:** Genes, forward and reverse primers for gene expression analysis by qRT-PCR.

Gene ^1^	5’-Primer Sequence Forward- 3´	5´-Primer Sequence Reverse- 3´	Ref.
UB	GGGATGCAGATCTTCGTGAAA	CTTGCCAGCAAAGATCAACCTT	[25]
β Actin	GTGATGGACTCTGGTGATGG	TGGTGAAGCTGTAGCCTCTC	[26]
TLR-2β	CGCTTAGGAGAGACAATCTGTGAA	GCCTGTTTTAGGGATTTCAGAGAATTT	[27]
TLR4	AGTCTGAAATTGCTGAGCTCAAAT	GCGACGTTAAGCCATGGAAG	[27]
TGF-β4	CGGCCGACGATGAGTGGCTC	CGGGGCCCATCTCACAGGGA	[27]
IL-8 (CXCLi2)	CCTGGTTTCAGCTGCTCTGT	GCGTCAGCTTCACATCTTGA	[28]
Bu-1	GGTGTCCAGTGAAGGTGTG	GATGCAAAGGATGGGTGTC	[29]
HSP-70	GGCTGGAGAGAAGAATGTGC	CAGCTGTGGACTTCACCTCA	[30]
Claudin1	TGGCCACGTCATGGTATGG	AACGGGTGTGAAAGGGTCATAG	[27]
Claudin3	GCCAAGATCACCATCGTCTC	CACCAGCGGGTTGTAGAAAT	[30]
FABP6	TGATTTCCCTGGACTCAGC	CCCACCTTCCATTTTGACTG	[31]
ChREBP	CTGAGCGATCGAAGGTGAA	TCTCCATCTTGCTGGAGTCA	[32]

^1^ UB, ubiquitin; TLR4, toll-like receptor 4; TLR-2β, toll-like receptor 2β; TGF-β4, transforming growth factor beta 4; IL-8, interleukin 8 (former CXCLi2); Bu-1, chicken B-cell marker chB6; HSP-70, heat shock protein 70; Claudin1, Claudin-1; Claudin3, Claudin-3; FABP6, fatty acid binding protein 6; ChREBP, carbohydrate-responsive element-binding protein.

**Table 3:** Effect of experimental diets on broiler chickens growth performance from 14 to 32 d of age ^1^.

Item ^4^	CNF	CF	OE500F	OE1500F	MF ^3^	SEM ^2^	*p*-Value		
							CNF vs. CF	OE Linear	OE No Linear
From 14 to 21 d									
ADG (g/bird/d)	72.4	68.9	70.0	69.4	71.7	1.24	0.06	0.80	0.61
ADFI (g/bird/d)	91.0	87.6	89.0	88.4	88.8	1.46	0.11	0.69	0.59
FCR (g/g)	1.26	1.27	1.27	1.28	1.24	0.012	0.49	0.79	0.96
From 21 to 32 d									
ADG (g/bird/d)	113	117	117	117	114	2.65	0.32	0.96	0.90
ADFI (g/bird/d)	165	170	169	168	164	3.48	0.28	0.62	0.95
FCR (g/g)	1.45	1.45	1.44	1.44	1.43	0.010	0.67	0.39	0.84

^1^ CNF, basal diet no fasted; CF, OE500F, OE1500F group, basal diet containing 0, 500 and 1500 ppm of olive pomace extract and fasted, respectively; MF, basal diet containing 100 ppm of monensin and fasted. ^2^ SEM, standard error of the mean (*n* = 6). ^3^ Fasted treatments were not significantly different to MF treatment by Dunnett’s test at 5%. ^4^ ADG, average daily gain; ADFI, average daily feed intake; FCR, feed conversion ratio.

**Table 4:** Effect of a short-term (15.5 h) fasting period and experimental diets on lactulose, mannitol and lactulose/mannitol ratio in chicken plasma at 15 d of age ^1^.

Item ^4^	CNF	CF	OE500F	OE1500F	MF ^3^	SEM ^2^	*p*-Value		
							CNF vs. CF	OE Linear	OE No Linear
Mannitol (µg/mL)	33.0	22.1	27.6	25.5	25.7	1.54	<0.001	0.13	0.053
Lactulose (µg/mL)	3.79	17.4	20.3	16.1	19.0	1.91	<0.001	0.62	0.13
L/M	0.11	0.76	0.73	0.64	0.69	0.065	<0.001	0.19	0.77

^1^ CNF, basal diet no fasted; CF, OE500F, OE1500F group, basal diet containing 0, 500 and 1500 ppm of olive pomace extract and fasted, respectively; MF, basal diet containing 100 ppm of monensin and fasted. ^2^ SEM, standard error of the mean (n = 12). ^3^ Fasted treatments were not significantly different to MF treatment by Dunnett’s test at 5%. ^4^ L/M, lactulose:mannitol ratio.

**Table 5:** Effect of a short-term (15.5 h) fasting period and experimental diets on AGP, IL-1β and IL-8 concentration in chicken plasma at 15 d of age ^1^.

Item ^4^	CNF	CF	OE500F	OE1500F	MF ^3^	SEM ^2^	*p*-Value ^5^		
							CNF vs. CF	OE Linear	OE No Linear
AGP (µg/mL)	191	**256**	221	217	186	15.7	0.006	0.081	0.43
IL-1β (ng/mL)	2.66	2.14	2.47	1.95	2.59	0.53	0.47	0.90	0.78
IL-8 (pg/mL)	69.1	52.9	19.2	46.0	43.3	16.8	0.76	0.80	0.46

^1^ CNF group, basal diet no fasted; CF, OE500F, OE1500F group, basal diet containing 0, 500 and 1500 ppm of olive pomace extract and fasted, respectively; MF, basal diet containing 100 ppm of monensin and fasted. ^2^ SEM, standard error of the mean (n = 12). ^3^ Values in bold in fasted treatments are significantly different to MF treatment by Dunnett’s test at 5%. ^4^ AGP, alpha-1-acid glycoprotein; IL-1β, interleukin 1β; IL-8, interleukin 8. ^5^ *p*-Values for IL-1 β and IL-8 are from root square data transformation analysis.

**Table 6:** Effect of a short-term (15.5 h) fasting period and experimental diets on ileum gene expression ^1^.

Item ^4^	CNF	CF	OE500F	OE1500F	MF ^3^	SEM ^2^	*p*-Value ^5^		
							CNF vs. CF	OE Linear	OE No Linear
Claudin1	10.6	11.4	11.6	11.5	11.5	0.24	0.031	0.73	0.68
Claudin3	7.39	7.43	7.42	7.46	7.47	0.16	0.86	0.88	0.89
ChREBP	11.0	11.9	11.8	11.6	11.5	0.35	0.063	0.50	0.94
FABP6	0.15	1.08	1.22	1.07	0.82	0.19	0.001	0.96	0.53
TLR-2β	7.89	8.14	7.89	7.69	7.90	0.21	0.40	0.13	0.91
TLR4	11.4	10.8	10.8	10.6	10.7	0.20	0.032	0.50	0.47
TGF-β4	13.7	13.4	13.4	13.5	13.4	0.16	0.19	0.66	0.63
IL-8	6.84	5.80	6.68	5.86	6.54	0.35	0.041	0.90	0.053
HSP-70	7.60	8.10	8.09	7.99	7.85	0.22	0.12	0.73	0.88
Bu-1	8.88	8.61	8.18	7.92	8.37	0.22	0.39	0.032	0.78

^1^ CNF group, basal diet no fasted; CF, OE500F, OE1500F group, basal diet containing 0, 500 and 1500 ppm of olive pomace extract and fasted, respectively; MF, basal diet containing 100 ppm of monensin and fasted. ^2^ SEM, standard error of the mean (*n* = 12). ^3^ Fasted treatments were not significantly different to MF treatment by Dunnett’s test at 5%. ^4^ Values are increments of cycle threshold (ΔCt) of target gene relative to the housekeeping. Ct values are inversely related to the RNA abundance. Claudin1, Claudin-1; Claudin3, Claudin-3; ChREBP, carbohydrate-responsive element-binding protein; FABP6, fatty acid binding protein 6; TLR-2β, toll-like receptor 2β; TLR4, toll-like receptor 4; TGF-β4, transforming growth factor beta 4; IL-8, interleukin 8 (former CXCLi2); HSP-70, heat shock protein 70; Bu-1, chicken B-cell marker chB6.

**Table 7:** Effect of a short-term (15.5 h) fasting period and experimental diets on villus height, crypt depth and villus/crypt ratio in broiler chickens duodenum at 15 d of age.^1.^

Item ^4^	CNF	CF	OE500F	OE1500F	MF ^3^	SEM ^2^	*p*-Value		
							CNF vs. CF	OE Linear	OE No Linear
Villus height (µm)	1956	**1907**	**1875**	1748	1726	45.2	0.44	0.019	0.40
Crypt depth (µm)	149	181	162	158	168	6.04	<0.001	0.014	0.33
Villus/Crypt	13.3	10.8	11.6	11.3	10.4	0.50	<0.001	0.45	0.34

^1^ CNF group, basal diet no fasted; CF, OE500F, OE1500F group, basal diet containing 0, 500 and 1500 ppm of olive pomace extract and fasted, respectively; MF, basal diet containing 100 ppm of monensin and fasted. ^2^ SEM, standard error of the mean (n = 12). ^3^ Values in bold in fasted treatments are significantly different to MF treatment by Dunnett’s test at 5%.
